# IL-30^†^ (IL-27A): a familiar stranger in immunity, inflammation, and cancer

**DOI:** 10.1038/s12276-021-00630-x

**Published:** 2021-05-28

**Authors:** Booki Min, Dongkyun Kim, Matthias J. Feige

**Affiliations:** 1 Department of Microbiology and Immunology, Northwestern University Feinberg School of Medicine, Chicago, IL 60611 USA; 2 Robert H. Lurie Comprehensive Cancer Center, Northwestern University Feinberg School of Medicine, Chicago, IL 60611 USA; 3 Department of Chemistry and Institute for Advanced Study, Technical University of Munich, 85748 Garching, Germany

**Keywords:** Interleukins, Cytokines

## Abstract

Over the years, interleukin (IL)-27 has received much attention because of its highly divergent, sometimes even opposing, functions in immunity. IL-30, the p28 subunit that forms IL-27 together with Ebi3 and is also known as IL-27p28 or IL-27A, has been considered a surrogate to represent IL-27. However, it was later discovered that IL-30 can form complexes with other protein subunits, potentially leading to overlapping or discrete functions. Furthermore, there is emerging evidence that IL-30 itself may perform immunomodulatory functions independent of Ebi3 or other binding partners and that IL-30 production is strongly associated with certain cancers in humans. In this review, we will discuss the biology of IL-30 and other IL-30-associated cytokines and their functions in inflammation and cancer.

## Immunity and cancer: A fresh look at a well-known signaling protein

Studying the ways that interleukin IL-30 regulates immune responses may provide novel insights into tumor development and inflammatory conditions. Interleukins are a diverse family of proteins involved in intercellular communications and immunity, where they can exert divergent and even opposing functions. Booki Min at Northwestern University in Chicago, USA, and co-workers reviewed the current understanding of IL-30 and its links to inflammation and cancer. IL-30 forms the IL-27 complex with the Ebi3 protein and was thought to be a surrogate for IL-27 in terms of activity. However, recent insights suggest that IL-30 may perform discrete immune modulation functions. Elevated IL-30 secretion is linked to prostate and breast cancer development. Extensive research is needed into the formation of IL-30, its associated protein interactions, and the development of a suitable animal model.

## Introduction

Adaptive T cell responses are shaped by multiple factors, and soluble mediators, especially cytokines produced by ‘innate’ immune cells, play an instructive role in the process^1^. Heterodimeric cytokines that belong to the IL-12 family are instrumental to the generation of inflammatory Th1 and Th17 immunity. The p35 and p19 subunits covalently linked to the common p40 subunit form bioactive IL-12 and IL-23, respectively, and are produced by activated antigen-presenting cells, such as macrophages and dendritic cells, following stimulation with innate stimuli via pattern recognition receptors, including TLRs^2^. These cytokines trigger differentiation processes in responding T cells through which the T cells acquire distinct effector properties conferring the ability to eliminate antigenic organisms. IL-12 activates the Stat4/T-bet pathway and generates IFNγ-producing Th1 effector cells capable of clearing intracellular pathogens, whereas IL-23 amplifies Th17 programs initiated through the IL-6-TGFβ-Rorγt axis, clearing extracellular bacterial or fungal infections^3,4^. The essential protective immunity mediated by these cytokines requires tight regulation, and inadequate control results in autoimmune inflammation. Targeting or utilizing these key cytokines and/or their signaling pathways is being exploited in therapeutic approaches to intervene in inflammatory disorders, including inflammatory bowel disease, psoriasis, and multiple sclerosis, and to develop cancer immunotherapies^2^.

IL-30, also known as IL-27p28 or IL-27A, was discovered in a search for novel helical cytokines^5^. It forms a noncovalent bond with the Ebi3 subunit, which also associates with the IL-12p35 subunit to form IL-35^6^. The IL-30/Ebi3 complex, named IL-27, exhibits anti-inflammatory (and regulatory) functions. It was initially thought to be an IL-12-like cytokine promoting Th1 immunity because of its ability to induce T-bet and IL-12Rβ2 expression through STAT1 activation during Th1 differentiation^7^. It was subsequently discovered that IL-27 also exerts a potent inhibitory role during Th17 differentiation^8^. It prevents activated T cells from acquiring RORγt and IL-17 expression via a STAT1-dependent mechanism^9^. IL-27 also suppresses Th2 immunity by inhibiting Th2 differentiation and cytokine production or by directly inhibiting group 2 innate lymphoid cells (ILC2s)^10,11^. IL-27 is a potent inducer of IL-10 in activated T cells but does not induce Foxp3 expression^12^. IL-27 also suppresses TGFβ-induced Foxp3^+^ regulatory T cell differentiation^13^, although it tends to support functions of already differentiated regulatory T cells^14,15^. Hence, IL-27 mediates an extremely broad array of functions involved in controlling T-cell immunity by targeting multiple pathways. Further extending the combinatorial complexity of the IL-12 family, there is increasing evidence that IL-30 can be secreted independently of Ebi3 and mediates distinct immunoregulatory functions^16^. In this review, we will discuss the immunobiology and functions of IL-30 and IL-30-associated cytokines (primarily IL-27) in the settings of inflammation and cancer (Table 1).

**Table 1:** Sources, targets, and functions of IL-27 and IL-30.

Cytokine complex	Source	Species	Target cells	Functions	Refs.
IL-27 and/or IL-30	Macrophage	M	T cells	Supports Th1 differentiation	^7^
				Inhibits Th2/Th17 differentiation	^8–11^
	Unknown	M, H		Induces IL-10 production	^12^
				Inhibits Treg differentiation	^13^
	Unknown	M	Treg	Enhances Treg suppressive function	^14,15^
	CD4 T cell	M	T cells	Inhibits IL-2 production and proliferation	^24^
IL-27	B cell	M	B cells	Supports proliferation, survival, and isotype switching Plasma cell differentiation	
IL-30^a^		M	CD8 T cells	Supports CD8 T cell reconstitution	^25^
IL-27	Epithelial cell Fibroblast	H	Fibroblast	Upregulates antiviral genes	^26^
IL-30^a^	Macrophage Neutrophil	M	γδ T cell Neutrophil	Suppresses γδ T cell activation and neutrophil expansion	^27^
IL-27	Neutrophil	H	Neutrophil	Reduces oxidative burst and antibacterial activity	^28^
IL-30	Macrophage	H	Cancer cell	Supports cancer stem-like cell survival	^92–94^

^a^In this study, only IL-30 expression was measured. Ebi3 expression was not determined.

## IL-30 and IL-27

The main source of IL-30 is cells of myeloid origin, namely, monocytes, macrophages, and dendritic cells^17^. IL-30 can also be produced by microglial cells and astrocytes in the central nervous system, alveolar macrophages and interstitial macrophages in lung tissue, and Kupffer cells in the liver^18–20^. Extensive efforts have been made to identify the cellular mechanisms inducing IL-30 expression. Molle et al. reported that TLR3 and TLR4 ligation in murine bone marrow-derived dendritic cells or human monocyte-derived dendritic cells triggers *Il30* (*Il27p28*) mRNA synthesis via IRF3-dependent mechanisms^21^. ELISA experiments measuring IL-27 production in this study utilized the IL-27p28/IL-30 Quantikine kit from the R&D Systems, which primarily measures IL-30 with modest cross-reactivity with recombinant IL-27 (Ebi3/p28 fusion protein) (https://resources.rndsystems.com/pdfs/datasheets/m2728.pdf). Thus, the cytokine measurements mostly represented IL-30 but also included the IL-27 heterodimer. Liu et al.^22^ reported that *Il30* (*Il27p28*) mRNA expression and IL-30 (IL-27p28) protein secretion (IL-27p28/IL-30 Quantikine ELISA kit from R&D Systems) in macrophages are induced through a MyD88-mediated activation pathway and that combined stimulation with IFNγ dramatically enhances the production. McNab et al. showed that type I IFN enhances IL-27 secretion in *M. tuberculosis*-infected macrophages^23^. The IL-27 ELISA kit from eBioscience (Thermo Fisher), which detects both forms of the cytokine and does not distinguish between the IL-27 heterodimer and IL-30, was used in this study. Therefore, stimulation via innate receptors or cytokines is the primary signal responsible for the generation of IL-30 and/or IL-27 in myeloid cells.

Kimura et al.^24^ unexpectedly discovered that a subset of murine CD4 T cells is also capable of secreting IL-30 and the IL-27 heterodimer. In this study, IL-30 secretion was measured using the Quantikine ELISA kit described above, while IL-27 heterodimer production was determined using the IL-27 LEGEND MAX^TM^ ELISA kit from BioLegend, which utilizes anti-IL-30 and anti-Ebi3 mAbs as the capture and detection Abs, respectively. The authors found that IL-27^+^ CD4 T cells exert a regulatory function, inhibiting the IL-2 production and proliferation of other T cells during malaria infection. These regulatory T cells are Foxp3^−^CD11a^+^CD49d^+^ antigen-specific cells and are distinct from Foxp3^+^ regulatory or IL-10-producing Tr1 T cells^24^. B cells also produce the IL-27 heterodimer in response to TLR and CD40 signaling, which was measured by sandwich ELISA using anti-IL-30 and anti-Ebi3 polyclonal Ab pairs (BioRXiv, doi.org/10.1101/2020.06.26.117010). IL-27^+^ B cells are not antibody-secreting cells but cooperate with IFNγ to support proliferation, survival, class switching, and plasma cell differentiation, functioning as ‘helper’ B cells that link innate and adaptive signals for optimal antibody responses. In another study, B cell-derived IL-30 was shown to be critical in promoting CD8 T cell reconstitution following antibody-mediated lymphoablation^25^. In a study using an antibody-mediated lymphoablation model in solid organ transplantation, it was discovered that CD8 T cell reconstitution requires CD4 T cells and B cell *Il30* mRNA expression^25^. In vivo neutralization using an anti-IL-30 mAb reversed CD8 T cell reconstitution^25^, although the precise nature of the cytokine (IL-30 vs. IL-27 heterodimer) involved in the process was not determined. Therefore, B cell-derived IL-27/30 may have important regulatory functions supporting adaptive B and T cell responses. In human uterine epithelial cells and fibroblasts, poly I:C stimulation induces IL-27 production, which was measured using human IL-27 ELISA Duoset from R&D Systems; this kit detects the IL-27 heterodimeric complex according to the manufacturer. Notably, the ELISA kit shows ~35% cross-reactivity with recombinant human Ebi3 (https://resources.rndsystems.com/pdfs/datasheets/dy2526.pdf). Whether it cross-reacts with recombinant human IL-30 is unclear. IL-27 appears to be involved in upregulating antiviral genes, including *OAS2* and *APOBEC4G*^26^. As estradiol modulates IL-27 production, this finding suggests that IL-27 may have an important immunoprotective role against incoming viral pathogens. In an IL-23-induced arthritis mouse model, neutrophils and macrophages were shown to produce IL-30, potentially suppressing arthritis^27^. Although *Il30* mRNA expression and IL-30 secretion (ELISA kit from R&D Systems) were measured^27^, Ebi3 expression was not determined. Therefore, whether IL-27 and/or IL-30 mediate immunomodulatory functions remains unknown. During sepsis caused by bacterial infections, neutrophils are an important source of IL-27^28^. Importantly, neutrophil-derived IL-30 can act directly on neutrophils, modulating bacteria-induced oxidative burst and cytokine production, i.e., antibacterial immunity^28^. The MaxDiscovery^TM^ IL-30 ELISA test kit from Bio Scientific was used to specifically measure IL-30 secretion in the neutrophil study^28^. Whether neutrophils produce the IL-27 complex or IL-30 during sepsis remains to be determined. Overall, IL-30 and IL-27 can be secreted by multiple sources, and the mechanisms inducing their production could be highly diverse. It is important to distinguish IL-30 from IL-27, as the former can exert biological functions in the absence of Ebi3 (discussed below). It is also worth noting that different reagents for IL-30 and or IL-27 detection may provide different readouts that are difficult to adequately interpret.

## Measuring IL-27 heterodimer production versus IL-30 production

While measuring the mRNA expression of p28 or p28-associated subunits is widely used to assess expression, it is equally important to assess protein complexes from different sources. Commercially available human IL-27 ELISA reagents measure IL-30/Ebi3 heterodimers, as human IL-30 is not freely secreted without Ebi3^29^. In the case of murine IL-27, however, extra precautions must be made when the reagents are chosen. Early studies reported IL-27 production by ELISA, which in fact measured IL-30 secretion as well. Murine IL-27 ELISA kits available from R&D Systems or Thermo Fisher will detect not only IL-27 complexes but also IL-30. For example, the DuoSet IL-30 ELISA kit from R&D Systems has 4.6% cross-reactivity with recombinant IL-30/Ebi3 proteins (the kit reads a sample containing 6.3 ng/ml recombinant mouse IL-30/Ebi3 as 286 pg/ml, https://resources.rndsystems.com/pdfs/datasheets/dy1834.pdf). Although the cross-reactivity of other IL-30 ELISA reagents is unknown, substantial cross-reactivity is highly suspected. There are separate reagents capable of detecting IL-30/Ebi3 only. An ELISA kit from BioLegend was designed to detect the IL-27 heterodimer. However, it needs to be determined whether free IL-30 is also detected. Alternatively, Pascual and colleagues successfully utilized a combination of anti-Ebi3 and anti-IL-30 mAbs to detect murine IL-27 complexes in culture supernatant^30^. We also utilized anti-Ebi3 (R&D Systems, cat# MAB18341) and biotinylated anti-IL-27 Abs (detection Ab included in the Thermo Fisher IL-27 ELISA kit, cat# 88-7274-88) as capture and detection Abs, respectively, to detect the IL-27 complex but not IL-30 (our unpublished result).

## Other IL-30 associated subunits

Another layer of complexity in IL-30 biology comes from its ability to form complexes with multiple subunits. In addition to the Ebi3 subunit, cytokine-like factor 1 (CLF1) can associate with IL-30. Crabe et al. reported that CLF1 encoded by the cytokine receptor-like factor 1 gene binds IL-30 and that the IL30/CLF1 heterodimer can be secreted by dendritic cells following activation^31^. The IL-30/CLF1 complex enhances NK cell activity, inhibits CD4 T cell proliferation, and induces IL-17 and IL-10 production, suggesting dendritic cell regulation of NK and T cell functions via this complex^31^. Tormo et al.^32^ demonstrated that the IL-30/CLF1 complex also acts on B cells. In particular, the complex induces enhanced antibody production and plasma cell differentiation. Interestingly, IL-27Rα is not necessary for the IL-30/CLF1 complex to control B cell functions. Murine IL-30 has also been shown to form a complex with the IL-12p40 subunit. Wang et al.^33^ utilized a bicistronic vector encoding both the p28 and p40 subunits and found that the two subunits could form a complex. The complex was shown to suppress T cell proliferation and differentiation into Th1/Th17 phenotype cells but support Treg expansion^33^. Flores et al. independently engineered IL-30/p40 complexes using a 15 amino acid linker and named them “IL-Y”^34^. IL-Y expresses potent anti-inflammatory properties; however, the effect appears to be independent of Tregs, as it reduces the proportion of Tregs^34^.

## Receptors and signaling of IL-27, IL-30, and IL-30-associated cytokines

As IL-30 is able to form distinct complexes with various partnering subunits, the receptors utilized by such divergent complexes are also variable. The receptor for IL-27 is a heterodimeric transmembrane protein composed of WSX-1 and gp130^5^. WSX-1, an IL-6/IL-12 family receptor, structurally resembles gp130^35^ and was initially thought to be necessary for the development of Th1-type immunity^36^. gp130 is shared by receptors for IL-6, IL-35, ciliary neurotrophic factor, and cardiotrophin-like cytokine, while WSX-1 expression determines IL-27 responsiveness and is thus named IL-27Rα. WSX-1 is highly expressed on lymphocytes, but other cell types including dendritic cells, macrophages, and intestinal epithelial cells also express the receptor^37–39^. IL-27 signaling utilizes the Jak/Stat and p38MAPK pathways^17,40^, and more than one Stat molecule is activated by IL-27 stimulation. Among the STATs, Stat1 and Stat3 are the primary mediators of IL-27 signaling. The highly diverse functions of IL-27 appear to utilize different Stat pathways. For example, Stat1 is essential for IL-27-induced T-bet expression, whereas IL-27-induced proliferation utilizes the Stat3 pathway^41,42^. The IL-27-Stat3 axis also plays a critical role in IL-27-induced PD-L1 or CD39 expression in macrophages and DCs^43,44^. On the other hand, the IL-27-Stat1 axis seems to function in IL-27-induced CD39 expression in regulatory T cells^45^. Therefore, IL-27-derived signaling pathways could differ depending on the target molecules and cell types.

The receptors utilized by IL-30/CLF1 complexes are somewhat distinct from the IL-30/Ebi3 pair. Utilizing Ba/F3 transfectants, Crabe et al.^31^ demonstrated that IL-30/CLF1 activates cells expressing IL-6Rα in addition to WSX-1, utilizing a tripartite receptor (gp130/WSX-1/IL-6Ra). In support of this idea, blocking IL-6R with an anti-IL-6Rα Ab is sufficient to inhibit IL-30/CLF1-induced proliferation^31^. Analogous to IL-27, IL-30/CLF1 activates both the Stat1 and Stat3 pathways. IL-30 signaling via IL-6Rα raises the possibility that IL-30 may associate with the soluble IL-6Rα protein to ‘trans-signal’ in target cells through gp130-expressing target cells^31^. Indeed, an IL-30/sIL-6Rα fusion protein was able to stimulate Stat3 pathways and to induce the proliferation of gp130 transfectants. However, whether this “trans-signaling” mechanism occurs during immune responses remains to be investigated.

There is emerging evidence that IL-30 is capable of exerting immunoregulatory functions independent of other subunits. Although the efficacy may be lower, IL-30 inhibits IL-12-induced liver toxicity as effectively as IL-27, independent of Ebi3 or IL-27Rα ^46^. During in vitro T cell differentiation, IL-30 is capable of inhibiting Th17 differentiation in the absence of Ebi3; however, unlike IL-27, IL-30 is unable to induce IL-10 expression^16^. Stumhofer et al. reported that IL-30 itself does not activate Stat molecules, although IL-30 does inhibit the Stat1 and Stat3 phosphorylation induced by IL-27^16^. However, these findings were observed in conditions where highly variable concentrations of recombinant IL-30 were used. For example, 50-100 ng of IL-30 is needed to antagonize IL-6-driven Stat3 phosphorylation or IL-27-driven Stat1 phosphorylation^47^. However, a study using transfection of gp130 and IL-6Rα showed that IL-30 induces phosphorylation of both Stat1 and Stat3 and that IL-30 can even activate signal transduction through gp130 without IL-6Rα at a higher concentration^47^. Likewise, a high concentration of human IL-30 can induce the phosphorylation of both Stat1 and Stat3^29^. Therefore, the precise ability of IL-30 to induce signaling demands further investigation.

## Murine models for investigating IL-27 and IL-30 biology

### IL-30 transgenic (Tg) mouse models

As an effort to understand the role of IL-30 in vivo, Stumhofer et al. generated IL-30 Tg mice in which IL-30 transgene expression is controlled by the lck proximal promoter and the immunoglobulin intronic heavy chain enhancer, driving IL-30 expression in B and T cells^16^. The IL-30 level in the serum of this animal model is 1–1.5 ng/ml. IL-30 overexpression in lymphocytes does not affect the number of mature B cells or the ratio of CD4 and CD8 T cells, although the total number of T cells with an activated phenotype is elevated. However, the production of inflammatory cytokines following in vitro stimulation was found to be comparable. No overt signs of tissue inflammation have been observed, and regulatory T cell development also remains intact. However, IL-30 overexpression results in the defective generation of antigen-specific IgG responses and germinal center formation. IL-30 Tg mice are resistant to autoimmune inflammation, EAE, and EAU, and the tissue infiltration of antigen-specific Th1 and Th17 cells is significantly reduced in this model^48^.

### IL-27 Tg mouse models

Nakanishi and colleagues generated IL-27 Tg mice overexpressing single-chain IL-27, in which Ebi3 is flexibly linked to IL-30 with the (Gly4Ser)3 linker, under the liver-specific human serum amyloid P component promoter and the rabbit b-globin gene^11^. The resulting Tg mice have a circulating serum IL-27 complex level of 0.5–1 ng and exhibit no obvious morphological abnormalities in the liver or immunological defects in the primary and secondary lymphoid tissues. Following induction of intestinal parasitic infection or allergic airway inflammation, IL-27 Tg mice display inhibited Th2 immune responses. However, these mice display dysregulated hematopoiesis characterized by increased megakaryocyte numbers and shortened survival^49^. A separate IL-27 Tg mouse model was generated by breeding mice expressing IL-30 and Ebi3 transgenes^50^. Transgene expression was limited to T and B cells. These IL-27 Tg mice spontaneously develop and succumb to systemic inflammatory disease, with extensive immune infiltrates found in multiple tissues. Peripheral immune cells display highly activated phenotypes and elevated inflammatory cytokine production. Importantly, Foxp3^+^ regulatory T cell development is severely defective in these IL-27 Tg mice, which is attributed to reduced IL-2 production.

### IL-27Rα-deficient mouse models

Germline IL-27Rα-deficient mice were developed by Mak and colleagues by targeting an exon encoding a part of the second fibronectin type III domain^51^. IL-27Rα deficiency does not affect hematopoietic or lymphocyte development. In vitro T cell proliferation is slightly increased, while IFNγ production is impaired. IL-27Rα^−/−^ mice display high susceptibility to *Leishmania major* infection. In support of this susceptibility, IFNγ production by KO T cells is greatly reduced. Separate IL-27Rα^−/−^ mice were also developed by Chen et al.^36^, in which exons 2–8 were targeted. Analogously, Th1-type cytokine production is greatly reduced in these KO mice. Defects in Th1 immunity were further tested in a *Listeria monocytogenes* infection model that relies on protective Th1 responses. Consistent with the in vitro findings, these KO mice were unable to clear the pathogens. We generated IL-27Rα conditional knockout animals to test the cell type-specific functions of IL-27 signaling^15^. When Foxp3^Cre^ transgenic mice were used in the cross to generate Treg-specific IL-27Rα^−/−^ mice, we found that IL-27 signaling in Tregs is indispensable for Treg-mediated inhibition of autoimmune and allergic inflammation^15,52^, suggesting that Tregs may be more critical than conventional T cells in mediating IL-27-dependent control of chronic inflammatory responses.

### IL-30-deficient mouse models

Germline IL-30^−/−^ mice were developed by Neurath and colleagues^53^. IL-30^−/−^ mice develop normally and do not display gross or histologic abnormalities. By using a T cell-dependent colitis model, IL-30^−/−^ mice were found to develop intestinal inflammation similar to that induced by wild-type T cells. On the other hand, IL-30 deficiency was associated with increased disease severity demonstrated by greater weight loss during intranasal Sendai virus infection^54^. This susceptibility was not due to defects in viral clearance but was associated with more severe pathology, suggesting that IL-30 or IL-27 is critical in limiting pathology during viral infection. An independent IL-30^−/−^ mouse model was also developed by Li and colleagues^55^. IL-30-deficient mice succumb to death following sublethal LPS injection. IL-30 conditional knockout mice were developed by Zhang et al.^56^. Dendritic cell-specific IL-30^−/^^−^ mice are highly susceptible to concanavalin A-induced hepatitis, which is attributed to uncontrolled production of IFNγ by CD4 T cells.

## IL-27 and IL-30 in inflammation

The ability of IL-27 to suppress inflammatory responses, particularly in autoimmunity, has extensively been investigated. The initial observation that IL-27Rα-deficient mice are highly susceptible to EAE strongly suggests that IL-27 plays a key role in regulating self-reactive Th17-type encephalitogenic T cells^57^. In support of this hypothesis, we and others have demonstrated that systemically administered IL-27 inhibits the development of EAE^15,58^. The primary target cells of IL-27 appear to be CD4 T cells, as Th17 differentiation is directly suppressed by IL-27^57^. However, we showed that IL-27 acting on Foxp3^+^ Treg cells might be more critical for the in vivo actions of IL-27. This conclusion was drawn by utilizing a Treg cell-specific IL-27Rα^−/−^ mouse model that develops severe EAE following induction. Given that the IL-27 responsiveness of conventional CD4 T cells remains intact in this model, it is intriguing to note that Treg stimulation by IL-27 is indispensable for IL-27-mediated regulation of autoimmune inflammatory responses. This notion is further supported by results from adoptive transfer experiments where only wild-type but not IL-27Rα^−/−^ Treg cells protected mice from lethal EAE when transferred into recipients whose endogenous Treg cells were depleted^59^.

Similar cellular mechanisms of IL-27 regulating inflammatory responses have also been observed in allergic inflammation. Intranasal administration of IL-27 reduces eosinophil infiltration in the airways and airway hyperresponsiveness as well as allergic rhinitis^60^. The IL-27-induced effects on T cell cytokine production and Treg cell expansion are most pronounced, suggesting that IL-27 may directly act on T cells^60^. Interestingly, Chen et al. reported that preventive administration of IL-27 was able to attenuate allergic inflammation but that therapeutic administration had little effect^61^. We also reported that the primary target cells for the anti-inflammatory functions of IL-27 are Foxp3^+^ Treg cells, since intranasal administration of IL-27 loses its effect in the absence of Treg cells or in Treg cell-specific IL-27Rα^−/^^−^ mice^52^. Human bronchial epithelial cells are known to express the IL-27 receptor complex^62^. Indeed, IL-27 enhances ICAM-1 expression on bronchial epithelial cells and augments TNFα-induced IL-6 production, which is partly attributed to the ability of IL-27 to increase TNFα receptor expression^62^.

It is worth noting that IL-27 may play divergent roles in different inflammatory diseases. The role of IL-27 in diabetes is different from that in other autoimmune diseases or allergic inflammation. Ciecko et al. reported that IL-27 is essential for type 1 diabetes development^63^. Utilizing NOD mice deficient in IL-30 or IL-27Rα, they found that these mice are completely resistant to diabetes development. The primary targets of IL-27 underlying this resistance seem to be T cells, as IL-27R^−^^/−^ splenic T cell transfer into NOD.Rag1^−^^/−^ recipients recapitulated resistance^63^. Mechanistically, IL-27 directly regulates the balance of Treg and Th1-type CD4 T cells, while it also modulates the pathogenic functions of CD8 T cells. Spontaneous development of autoimmune inflammation in the lacrimal and salivary glands in NOD mice is similarly affected by IL-27^63^. Interestingly, Qi et al.^64^ recently reported contrary findings for IL-27 in experimental Sjögren’s syndrome. Utilizing the same IL-30^−/−^ NOD mouse model, it was reported that IL-30 deficiency results in aggravated disease manifestation and that severe disease is associated with a reduction in IL-10^+^ CD4 T cell subsets. Exogenous IL-27 administration attenuates inflammation and increases IL-10^+^ CD4 cells. In support of this, the levels of plasma IL-27 and IL-10^+^ CD4 T cells are decreased in patients with Sjögren’s syndrome^64^.

IL-27 induces the inhibitory receptor T cell immunoglobulin and mucin domain-3 (Tim-3) and IL-10 via a nuclear factor, NFIL3^65^. NFIL3-overexpressing CD4 T cells attenuate T cell-induced colitis, and elevated IL-10/Tim-3 and reduced IFNγ expression are found in these T cells. Furthermore, IL-27 treatment fails to suppress inflammation in NFIL3-deficient T cells.

A potent anti-inflammatory function of IL-30 was first reported in a cytokine-induced liver injury model. Li and colleagues reported that IL-30 expression was induced by inflammatory cytokines such as IL-12 and IFNγ and that IL-30 administered via hydrodynamic delivery of a plasmid encoding IL-30 attenuated liver fibrosis induced by CCl_4_ or hepatotoxicity induced by IL-12 independent of IL-27 or IL-27Rα^46,66^. Mechanistically, IL-30 inhibits hepatotoxicity by reducing the IFNγ level^46^. Moreover, the IL-30-induced reduction in liver fibrosis occurs via the removal of activated hepatic stellate cells by NK cells recruited to the liver^66^. Another approach for systemic IL-30 delivery using adeno-associated viral vectors (AAVs) found that systemic IL-30 delivery only slightly inhibited EAE development^67^. Of note, the serum IL-30 level measured following AAV-IL-30 administration was maintained at ~200 ng/ml^67^, whereas the level following hydrodynamic plasmid delivery was only ~1 ng/ml^66^. Whether different serum IL-30 levels underlie the discrepant findings remains to be examined. IL-30 has also been shown to attenuate experimental sepsis by modulating NKT cell cytokine profiles^55^. Caspi and colleagues utilized IL-30 Tg mice and reported that IL-30-overexpressing mice are highly resistant to autoimmune inflammation, EAE, and EAU (experimental autoimmune uveitis), primarily by antagonizing Th1 and Th17 responses^48^. Whether this is due to IL-30 or to the elevated formation of the IL-27 complex remains unclear.

## IL-27 and IL-30 in infection

The role of IL-27 was first tested in a *Toxoplasma gondii* infection model, in which protective immunity is dependent on the development of Th1-type immunity. Villarino et al. reported that infected IL-27Rα^−/−^ mice succumbed to infection due to uncontrolled T cell-mediated inflammatory responses^68^. The parasite burden was effectively controlled; however, T cells in this condition displayed a highly activated phenotype with increased proliferative activity^68^. Similar IL-27-dependent regulation of CD4 T cell immunity was also noted in an African trypanosome infection model^69^. Montes de Oca et al. recently reported a similar finding in a Leishmania infection model, in which IL-27R deficiency significantly expedited parasite clearance at the expense of enhanced tissue damage caused by an imbalance in antigen-specific Th1- and Tr1-type CD4 T cells^70^. Interestingly, IL-27 exerted these effects by regulating the metabolic profiles of Th1-type CD4 T cells^70^. IL-27Rα^−/−^ CD4 T cells were more glycolytic, suggesting that IL-27 seems to limit Th1 cell glycolysis to further protect against tissue pathology during infection.

The role of IL-27 was further explored in viral infections. Zuniga and colleagues reported a T cell-intrinsic role for IL-27 in chronic viral infection^71^. IL-27R deficiency in T cells impairs the accumulation of virus-specific CD4 T cells and the control of the viral load during chronic LCMV infection. Moreover, IL-27 signaling has been shown to control key innate immune cells during early LCMV infection, as type I IFN expression by DCs and NK cell function indicated by granzyme and IFNγ production seem to be affected by IL-27R deficiency^72^. IL-27 signaling is also important for the generation of IFNγ-producing CD8 T cells in infection. Mohrs and colleagues reported that in both *T. gondii* and influenza virus infection models, IL-27Rα^−/−^ CD8 T cells are defective in IFNγ production^73^.

In addition to CD4 and CD8 T cells, different target cells of IL-27 have been identified in other infection models. Moro et al. reported that IL-27 antagonizes the function of ILC2s using an *Alternaria alternata*-induced lung inflammation model^74^. *A. alternata* is a major fungus associated with ILC2-mediated asthmatic inflammation. Coadministration of *A. alternata* extract with IL-27 results in significantly reduced eosinophilia and type 2 cytokine production in the lungs. IL-27 reduces the production of eotaxin and type 2 cytokines by ILC2s but not by Th2 CD4 T cells. In support of this finding, IL-27Rα expression in ILC2s is greater than that in CD4 T cells, suggesting that IL-27 may have different impacts on ILC2s versus T cells^74^. Mchedlidze et al. demonstrated that IL-27 is able to suppress antihelminth immunity by directly targeting ILC2 responses^10^. More recently, Kwock et al.^75^ reported IL-27 signaling in skin cells during Zika virus infection. Treatment of human epidermal keratinocytes with IL-27 induces antiviral proteins such as oligoadenylate synthase (OAS), MX1, IRF1, and MDA5. Indeed, IL-27-induced antiviral proteins are functional, as IL-27 treatment substantially suppresses Zika virus infection in human keratinocytes. Moreover, IL-27 inhibits Zika virus morbidity and mortality following subcutaneous infection^75^.

Whether IL-30 plays a regulatory role in the setting of infection has not been formally explored.

## IL-27 and IL-30 in cancer

Divergent roles for IL-27 and IL-30 in anticancer immunity are being increasingly appreciated. Because of the abilities of IL-27 to induce IFNγ and activate NK cell activity, the antitumor function of IL-27 has been examined. Utilizing single-chain IL-27-secreting colon carcinoma and murine neuroblastoma models, it was shown that these tumor cells induce robust antitumor immune responses^76,77^. IFNγ neutralization or CD8 T cell depletion abrogates these antitumor effects, suggesting a link between IL-27 and adaptive cytotoxic immunity^76,77^. In NK cells, IL-27 directly activates T-bet and granzyme B expression and enhances both viability and cytolytic activity^78^. IL-27 gene transfer results in rejection of NK cell-resistant head and neck squamous cell carcinoma by NK cells^78^. Alternatively, IL-27 can support hematopoietic stem cell differentiation into M1-type macrophages and enhance their antitumor effects. Yoshimoto and colleagues reported that engineering B16F10 melanoma cells to express IL-27 substantially increased the number of tumor-infiltrating CD11b^+^ myeloid cells with expression of M1-type genes including iNOS, IRF8, and Il12p40 and decreased expression of M2-associated markers such as Arg-1, Ym1, and Fizz1^49,79^. The importance of myeloid cells was further corroborated by anti-Gr1 Ab treatment, which abrogated the antitumor effects of IL-27^79^. IL-27 is also able to directly act on tumor cells to perform antitumor functions. IL-27 inhibits the proliferation of B16 melanoma transfectants expressing IL-27R by augmenting the expression of IRF-1^80^. It was further demonstrated that IL-27 has broad impacts on the expression of genes involved in angiogenesis and invasion. Zorzoli et al.^81^ reported that IL-27 inhibits acute myeloid leukemia (AML) cell growth in an NSG mouse model by upregulating antiangiogenic genes such as IFNγ, CXCL10, and tissue inhibitors of metalloproteinase (TIMP)-2 and by downregulating genes involved in tumor spreading such as cadherin-6 (CDH6). Similar antiproliferative properties of IL-27 were also observed in prostate cancer cells. Di Carlo et al. reported that IL-27 inhibits prostate cancer cell proliferation and modulates the expression of genes involved in the angiogenic process such as fms-related tyrosine kinase, fibroblast growth factor receptor, CXCL10, and TIMP3^82^. Different IL-27 target genes were also identified in primary human multiple myeloma cells^83^. Incubation of cancer cells with IL-27 results in downregulation of proangiogenic genes, including AKT, angiopoietin, MMP9, VEGF, and laminin, and in upregulation of antiangiogenic genes, such as CXCL9 and CXCL10^83^. IL-27 also downregulates multiple stemness-related genes in lung adenocarcinoma and squamous cell carcinoma cell lines and inhibits tumor cell growth in xenograft models^84^. Last, IL-27 can inhibit human melanoma cell growth by inducing the expression of TNF-related apoptosis-inducing ligand^85^. Overall, IL-27 appears to exert antitumor effects by targeting multiple genes and pathways. It is worth noting that IL-27R expression (i.e., an indicator of IL-27 responsiveness) is lost in high-grade and advanced-stage prostate cancer cells^82^. Since tumor-infiltrating immune cells express functional IL-27R, harnessing the ability of IL-27 to stimulate antitumor activity in immune cells could be an effective approach.

However, the protumorigenic functions of IL-27 have also been observed. As discussed above, IL-27 is a potent inducer of IL-10 in T cells, and IL-10 is capable of inhibiting antitumor immunity^86^. Karakhanova et al. reported that IL-27 induces PD-L1 expression on DCs and that IL-27-treated DCs display a reduced capacity to stimulate T cell proliferation and cytokine production^87^. IL-27 acting on DCs has an additional impact, negatively regulating T cell immunity. It was shown that IL-27 induces CD39 expression in DCs and that IL-39 reduces extracellular ATP and diminishes nucleotide-dependent activation of the inflammasome, resulting in T cell inhibition^44^. The protumor activity of IL-27 may also be mediated by Treg cells. We previously reported that IL-27 stimulation of Treg cells is necessary for Treg cells to properly control T cell responses^15^. Park et al.^45^ recently reported that IL-27 stimulation induces CD39 expression on tumor-infiltrating Treg cells, leading to inhibition of CD8 T cell activation. IL-27R deficiency in Treg cells is thus sufficient to rescue antitumor CD8 T cell activity from Treg cell-mediated inhibition^45^. In addition, IL-27 may induce immune inhibitory molecules in human epithelial ovarian cancer cells, including the IL-18 binding protein, an inhibitor of IL-18 proinflammatory activity^88^; indoleamine 2,3-dioxygenase (IDO), which depletes tryptophan and causes T cell dysfunction and death^89^; and PD-L1, which inactivates T cell function by engaging PD-1 on activated T cells^90^. Finally, IL-27 also directly promotes the proliferation and survival of AML cells^91^. This pro-proliferative/survival effect of IL-27 seems to operate via the MAPK/ERK signaling pathway. IL-27 is also capable of interfering with the responsiveness of cancer cells to the chemotherapeutic drugs cytarabine and daunorubicin^91^.

In contrast, the roles of IL-30 are mostly protumorigenic. Di Meo et al. reported for the first time that IL-30 may support prostate cancer development^92^. From immunohistochemistry and real-time RT-PCR analyses, elevated IL-30 expression was found in prostate cancer epithelial cells and seemed to be correlated with an advanced grade and stage^92^. IL-30 expression was most pronounced in tumor-infiltrating leukocytes, particularly CD68^+^ macrophages, and was found even in infiltrating leukocytes in the prostate-draining lymph node. The mechanism underlying the action of IL-30 on prostate cancer progression may be linked to the ability of IL-30 to support prostate cancer stem-like cell (PCSLC) survival^93^. PCSLCs are able to produce IL-30 and promote tumor onset by enhancing proliferation and vascularization. Airoldi et al.^94^ reported that IL-30 expression is similarly elevated in breast cancer patients, particularly those with triple-negative HER2^+^ subtypes, and that analogous to prostate cancers, IL-30 is found in inflammatory leukocytes infiltrating tumors and the draining lymph nodes, and its expression correlates with the stage of the disease. Mechanistically, IL-30 alters the PCSLC expression of genes involved in immune suppression, stemness, and metastasis. IL-30 also increases the expression of the proto-oncogene *Myc* and *Muc1*, along with that of the genes encoding the growth factors EGF and VEGF-A^94^. In a xenograft model system, local IL-30 injection into mice implanted with breast cancer cells substantially upregulated genes involved in tumor growth and angiogenesis^94^.

## Disparities in IL-30 biology between mice and men

**Fig. 1:**
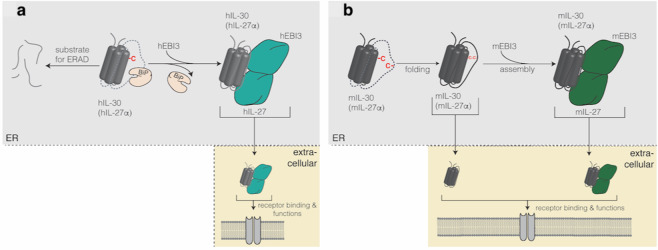
Species differences in IL-30 and IL-27 biogenesis. **a** The biogenesis of human (h) IL-27 is shown. Human IL-30 depends on assembly with Ebi3 to fold, be released from the endoplasmic reticulum (ER) chaperone Bip and function once outside the cell. If it remains unpaired, it is degraded by ER-associated degradation (ERAD). **b** In contrast, murine (m) IL-30 has two structurally adjacent cysteines **c** that can form a disulfide bond, so murine IL-30 can fold and be secreted in isolation or as a pair with Ebi3.

Most of our insights into IL-30 biology stem from mouse models, but analysis of human and mouse IL-30 reveals an important disparity in the secretion pattern of this cytokine. Mouse IL-30 is readily secreted by various cell types, immune cells, and transfected model cell lines into the culture supernatant in the absence of Ebi3. Coexpression of Ebi3 further enhances the secretion of IL-30 as part of the heterodimeric IL-27 complex^29,95^. In contrast, human IL-30 is not secreted in isolation but instead retained in the endoplasmic reticulum of cells, where it becomes a target of ER-associated degradation, showing that human IL-30 does not fold properly in isolation^29^ (Fig. 1). Coexpression of Ebi3 induces secretion of human IL-30^95^, and it has recently been shown that human IL-30 and Ebi3 indeed form a stable complex, IL-27, so that cellular retention of Ebi3 coretains IL-30 in cells^29^. This discrepancy was attributed to a single amino acid difference between the species. Mouse IL-30 has two cysteines, which can form a disulfide bond to stabilize the protein. The first cysteine residue is located in helix B of the four-helix bundle fold of IL-30, and the second cysteine residue is located in a large flexible loop connecting the C and D helices^96,97^. Human IL-30 has a leucine at the position corresponding to the second cysteine^96,97^. Importantly, the substitution of this leucine with a cysteine (L162C) in human IL-30 is sufficient to render it secretion competent in isolation regardless of Ebi3 expression, similar to murine IL-30^29^. Along the same line, mutation of the second cysteine in murine IL-30 (C158L) renders its secretion dependent on the coexpression of Ebi3^29^. Detailed computational and biochemical analyses have provided insights into the underlying mechanism: disulfide bond formation in IL-30 leads to the shielding of hydrophobic residues, enabling escape from the ER Hsp70 chaperone BiP for autonomous secretion^29^. A species comparison revealed that this mechanism is strictly conserved in evolution: whenever IL-30 forms a disulfide bond, it can be secreted autonomously, becoming capable of performing biological functions; if it fails to form a disulfide bond, e.g., due to having only a single cysteine, its secretion depends on Ebi3^96^. Mice and humans are thus only representatives of a larger number of species in regard to IL-30 secretion. This finding is of particular interest since cysteines are among the most highly evolutionarily conserved amino acids^98^, implying functional selection criteria during evolution that have favored the presence or absence of secretion-competent IL-30. IL-12 heterodimers, including IL-27, have likely evolved from cytokine:receptor pairs^99^. During evolution, in certain species, including humans, IL-27 has evolved not only into a cytokine but also into an obligate heterodimeric cytokine by losing one of the cysteines in the IL-30 subunit. Although humans thus seem to have generally lost secretion of IL-30 during evolution, human cells remain responsive to this cytokine^29^, which potentially opens up doors for using engineered human IL-30 for immune modulation. Furthermore, the multiple pairings of IL-30 and roles for IL-30 in human immunity may suggest that we do not appreciate the full picture of human IL-30 secretion and functions yet. As an example, polymorphisms in the IL-30 gene have been reported in numerous disease settings, including premature coronary artery disease^100^, bladder cancer^101^, osteosarcoma^102^, asthma^103^, autoimmune thyroid disease^104^, and IBD^105^. Whether these mutations alter the folding and secretion pattern of IL-30 or one of its pairings remains to be determined.

## Conclusion and outstanding questions

Given the emerging evidence for the ability of IL-30 to regulate immune responses by itself, it is evident that IL-30 is more than a partnering subunit that forms IL-27 complexes. Here, outstanding questions demanding future investigations are discussed. First, the formation of various IL-30-associated complexes, their precise sources and tissue sites, and the mechanisms involved in their production need to be examined. It is possible that different sources of myeloid cell origin may be the primary sources of IL-30-associated cytokine complexes and that the nature of inflammatory cues and tissue factors may play a role. Second, reagents specific for IL-30 and IL-30-associated complexes need to be developed. As discussed above, one cannot distinguish the production of IL-30 from that of complexes containing IL-30. ELISA reagents that specifically detect IL-30/Ebi3 complexes but not IL-30 are now available and are beginning to be used. Because of considerable cross-reactivity, however, detecting IL-30 free of other binding partners remains challenging. Third, based on the disparity between murine and human IL-30 secretory behavior, it is important to develop an animal model where IL-30 behaves like human IL-30, i.e., lack of secretion of IL-30 in the absence of binding proteins. This model could be useful for gaining insights into the functions of IL-30 in vivo. Fourth, elevated IL-30 secretion is strongly associated with numerous types of cancer, raising the possibility that dysregulated IL-30 secretion may be linked to cancer development. Therefore, it is important to understand the causal relations among IL-30 polymorphisms; IL-30 folding, secretion, and pairing patterns; and the progression of diseases, including tumors and other inflammatory conditions.

## Note

^†^IL27 (also known as IL30, IL-27A, p28, or IL27p28) is an approved name for the interleukin-27 alpha subunit (HUGO Gene Nomenclature Committee). In this review paper, we will use *IL30* (*Il30*) and IL-30 for the gene and protein product, respectively.

## References

[1] Iwasaki A, Medzhitov R (2015). Control of adaptive immunity by the innate immune system. Nat. Immunol..

[2] Teng MW (2015). IL-12 and IL-23 cytokines: from discovery to targeted therapies for immune-mediated inflammatory diseases. Nat. Med..

[3] Mullen AC (2001). Role of T-bet in commitment of TH1 cells before IL-12-dependent selection. Science.

[4] Gaffen SL, Jain R, Garg AV, Cua DJ (2014). The IL-23-IL-17 immune axis: from mechanisms to therapeutic testing. Nat. Rev. Immunol..

[5] Pflanz S (2002). IL-27, a heterodimeric cytokine composed of EBI3 and p28 protein, induces proliferation of naive CD4+ T cells. Immunity.

[6] Egwuagu CE, Yu CR, Sun L, Wang R (2015). Interleukin 35: Critical regulator of immunity and lymphocyte-mediated diseases. Cytokine Growth Factor Rev..

[7] Takeda A (2003). Cutting edge: role of IL-27/WSX-1 signaling for induction of T-bet through activation of STAT1 during initial Th1 commitment. J. Immunol..

[8] McAleer JP, Kolls JK (2011). Mechanisms controlling Th17 cytokine expression and host defense. J. Leukoc. Biol..

[9] Diveu C (2009). IL-27 blocks RORc expression to inhibit lineage commitment of Th17 cells. J. Immunol..

[10] McHedlidze T (2016). IL-27 suppresses type 2 immune responses in vivo via direct effects on group 2 innate lymphoid cells. Mucosal Immunol..

[11] Yoshimoto T, Yoshimoto T, Yasuda K, Mizuguchi J, Nakanishi K (2007). IL-27 suppresses Th2 cell development and Th2 cytokines production from polarized Th2 cells: a novel therapeutic way for Th2-mediated allergic inflammation. J. Immunol..

[12] Batten M (2008). Cutting edge: IL-27 is a potent inducer of IL-10 but not FoxP3 in murine T cells. J. Immunol..

[13] Neufert C (2007). IL-27 controls the development of inducible regulatory T cells and Th17 cells via differential effects on STAT1. Eur. J. Immunol..

[14] Hall AO (2012). The cytokines interleukin 27 and interferon-gamma promote distinct Treg cell populations required to limit infection-induced pathology. Immunity.

[15] Do J (2017). Treg-specific IL-27Ralpha deletion uncovers a key role for IL-27 in Treg function to control autoimmunity. Proc. Natl Acad. Sci. USA.

[16] Stumhofer JS (2010). A role for IL-27p28 as an antagonist of gp130-mediated signaling. Nat. Immunol..

[17] Yoshida H, Hunter CA (2015). The immunobiology of interleukin-27. Annu. Rev. Immunol..

[18] Senecal V (2016). Production of IL-27 in multiple sclerosis lesions by astrocytes and myeloid cells: Modulation of local immune responses. Glia.

[19] Mathie SA (2015). Alveolar macrophages are sentinels of murine pulmonary homeostasis following inhaled antigen challenge. Allergy.

[20] Bartkowiak T (2018). Activation of 4-1BB on liver myeloid cells triggers hepatitis via an interleukin-27-dependent pathway. Clin. Cancer Res..

[21] Molle C (2007). IL-27 synthesis induced by TLR ligation critically depends on IFN regulatory factor 3. J. Immunol..

[22] Liu J, Guan X, Ma X (2007). Regulation of IL-27 p28 gene expression in macrophages through MyD88- and interferon-gamma-mediated pathways. J. Exp. Med..

[23] McNab FW (2014). Type I IFN induces IL-10 production in an IL-27-independent manner and blocks responsiveness to IFN-gamma for production of IL-12 and bacterial killing in Mycobacterium tuberculosis-infected macrophages. J. Immunol..

[24] Kimura D (2016). Interleukin-27-producing CD4(+) T cells regulate protective immunity during malaria parasite infection. Immunity.

[25] Ayasoufi, K. et al. Interleukin-27 promotes CD8+ T cell reconstitution following antibody-mediated lymphoablation. *JCI Insight*10.1172/jci.insight.125489 (2019). 10.1172/jci.insight.125489 PMC6483639 30944247

[26] Patel MV, Shen Z, Rossoll RM, Wira CR (2018). IL-27 expression and responsiveness in human uterine epithelial cells and fibroblasts in vitro and the role of estradiol. J. Interferon Cytokine Res..

[27] Bouchareychas L, Grossinger EM, Kang M, Adamopoulos IE (2018). gammadeltaTCR regulates production of interleukin-27 by neutrophils and attenuates inflammatory arthritis. Sci. Rep..

[28] Rinchai D (2012). Production of interleukin-27 by human neutrophils regulates their function during bacterial infection. Eur. J. Immunol..

[29] Muller SI (2019). A folding switch regulates interleukin 27 biogenesis and secretion of its alpha-subunit as a cytokine. Proc. Natl Acad. Sci. USA.

[30] Kochetkova I (2014). Oral Escherichia coli colonization factor antigen I fimbriae ameliorate arthritis via IL-35, not IL-27. J. Immunol..

[31] Crabe S (2009). The IL-27 p28 subunit binds cytokine-like factor 1 to form a cytokine regulating NK and T cell activities requiring IL-6R for signaling. J. Immunol..

[32] Tormo AJ (2013). The composite cytokine p28/cytokine-like factor 1 sustains B cell proliferation and promotes plasma cell differentiation. J. Immunol..

[33] Wang RX, Yu CR, Mahdi RM, Egwuagu CE (2012). Novel IL27p28/IL12p40 cytokine suppressed experimental autoimmune uveitis by inhibiting autoreactive Th1/Th17 cells and promoting expansion of regulatory T cells. J. Biol. Chem..

[34] Flores RR (2015). IL-Y, a synthetic member of the IL-12 cytokine family, suppresses the development of type 1 diabetes in NOD mice. Eur. J. Immunol..

[35] Sprecher CA (1998). Cloning and characterization of a novel class I cytokine receptor. Biochem. Biophys. Res. Commun..

[36] Chen Q (2000). Development of Th1-type immune responses requires the type I cytokine receptor TCCR. Nature.

[37] Peshkova IO, Fatkhullina AR, Mikulski Z, Ley K, Koltsova EK (2017). IL-27R signaling controls myeloid cells accumulation and antigen-presentation in atherosclerosis. Sci. Rep..

[38] Visperas A, Do JS, Bulek K, Li X, Min B (2014). IL-27, targeting antigen-presenting cells, promotes Th17 differentiation and colitis in mice. Mucosal Immunol..

[39] Diegelmann J, Olszak T, Goke B, Blumberg RS, Brand S (2012). A novel role for interleukin-27 (IL-27) as mediator of intestinal epithelial barrier protection mediated via differential signal transducer and activator of transcription (STAT) protein signaling and induction of antibacterial and anti-inflammatory proteins. J. Biol. Chem..

[40] Sharma G (2014). IL-27 inhibits IFN-gamma induced autophagy by concomitant induction of JAK/PI3 K/Akt/mTOR cascade and up-regulation of Mcl-1 in Mycobacterium tuberculosis H37Rv infected macrophages. Int. J. Biochem. Cell Biol..

[41] Kamiya S (2004). An indispensable role for STAT1 in IL-27-induced T-bet expression but not proliferation of naive CD4+ T cells. J. Immunol..

[42] Owaki T (2008). STAT3 is indispensable to IL-27-mediated cell proliferation but not to IL-27-induced Th1 differentiation and suppression of proinflammatory cytokine production. J. Immunol..

[43] Horlad H (2016). An IL-27/Stat3 axis induces expression of programmed cell death 1 ligands (PD-L1/2) on infiltrating macrophages in lymphoma. Cancer Sci..

[44] Mascanfroni ID (2013). IL-27 acts on DCs to suppress the T cell response and autoimmunity by inducing expression of the immunoregulatory molecule CD39. Nat. Immunol..

[45] Park YJ (2019). IL-27 confers a protumorigenic activity of regulatory T cells via CD39. Proc. Natl Acad. Sci. USA.

[46] Dibra D (2012). Interleukin-30: a novel antiinflammatory cytokine candidate for prevention and treatment of inflammatory cytokine-induced liver injury. Hepatology.

[47] Garbers C (2013). An interleukin-6 receptor-dependent molecular switch mediates signal transduction of the IL-27 cytokine subunit p28 (IL-30) via a gp130 protein receptor homodimer. J. Biol. Chem..

[48] Chong WP (2014). IL-27p28 inhibits central nervous system autoimmunity by concurrently antagonizing Th1 and Th17 responses. J. Autoimmun..

[49] Seita J (2008). Interleukin-27 directly induces differentiation in hematopoietic stem cells. Blood.

[50] Wojno ED (2011). A role for IL-27 in limiting T regulatory cell populations. J. Immunol..

[51] Yoshida H (2001). WSX-1 is required for the initiation of Th1 responses and resistance to L. major infection. Immunity.

[52] Nguyen, Q. T. et al. IL-27 targets Foxp3+ Tregs to mediate antiinflammatory functions during experimental allergic airway inflammation. *JCI Insight*10.1172/jci.insight.123216 (2019). 10.1172/jci.insight.123216 PMC6413774 30674714

[53] Wirtz S, Billmeier U, McHedlidze T, Blumberg RS, Neurath MF (2011). Interleukin-35 mediates mucosal immune responses that protect against T-cell-dependent colitis. Gastroenterology.

[54] Muallem G (2017). IL-27 limits type 2 immunopathology following parainfluenza virus infection. PLoS Pathog..

[55] Yan J (2016). Interleukin-30 (IL27p28) alleviates experimental sepsis by modulating cytokine profile in NKT cells. J. Hepatol..

[56] Zhang S (2013). High susceptibility to liver injury in IL-27 p28 conditional knockout mice involves intrinsic interferon-gamma dysregulation of CD4+ T cells. Hepatology.

[57] Batten M (2006). Interleukin 27 limits autoimmune encephalomyelitis by suppressing the development of interleukin 17-producing T cells. Nat. Immunol..

[58] Fitzgerald DC (2007). Suppressive effect of IL-27 on encephalitogenic Th17 cells and the effector phase of experimental autoimmune encephalomyelitis. J. Immunol..

[59] Kim D (2019). Cutting Edge: IL-27 Attenuates Autoimmune Neuroinflammation via Regulatory T Cell/Lag3-Dependent but IL-10-Independent Mechanisms In Vivo. J. Immunol..

[60] Suzuki M, Yokota M, Ozaki S, Matsumoto T (2019). Intranasal Administration of Il-27 Ameliorates Nasal Allergic Responses and Symptoms. Int. Arch. Allergy Immunol..

[61] Su X (2016). IL-27 attenuates airway inflammation in a mouse asthma model via the STAT1 and GADD45gamma/p38 MAPK pathways. J. Transl. Med.

[62] Cao J, Wong CK, Yin Y, Lam CW (2010). Activation of human bronchial epithelial cells by inflammatory cytokines IL-27 and TNF-alpha: implications for immunopathophysiology of airway inflammation. J. Cell Physiol..

[63] Ciecko AE (2019). Interleukin-27 is essential for type 1 diabetes development and Sjogren syndrome-like inflammation. Cell Rep..

[64] Qi J (2020). IL-27 regulated CD4(+)IL-10(+) T cells in experimental Sjogren syndrome. Front. Immunol..

[65] Zhu C (2015). An IL-27/NFIL3 signalling axis drives Tim-3 and IL-10 expression and T-cell dysfunction. Nat. Commun..

[66] Mitra A (2014). IL-30 (IL27p28) attenuates liver fibrosis through inducing NKG2D-rae1 interaction between NKT and activated hepatic stellate cells in mice. Hepatology.

[67] Zhu J (2018). Interleukin-27 gene therapy prevents the development of autoimmune encephalomyelitis but fails to attenuate established inflammation due to the expansion of CD11b(+)Gr-1(+) myeloid cells. Front. Immunol..

[68] Villarino A (2003). The IL-27R (WSX-1) is required to suppress T cell hyperactivity during infection. Immunity.

[69] Liu G (2015). IL-27 signaling is crucial for survival of mice infected with african trypanosomes via preventing lethal effects of CD4+ T cells and IFN-gamma. PLoS Pathog..

[70] Montes de Oca M (2020). IL-27 signalling regulates glycolysis in Th1 cells to limit immunopathology during infection. PLoS Pathog..

[71] Harker JA, Dolgoter A, Zuniga EI (2013). Cell-intrinsic IL-27 and gp130 cytokine receptor signaling regulates virus-specific CD4(+) T cell responses and viral control during chronic infection. Immunity.

[72] Harker, J. A. et al. Interleukin-27R signaling mediates early viral containment and impacts innate and adaptive immunity after chronic lymphocytic choriomeningitis virus infection. *J. Virol.*10.1128/JVI.02196-17 (2018). 10.1128/JVI.02196-17 PMC5974502 29593047

[73] Mayer KD (2008). Cutting edge: T-bet and IL-27R are critical for in vivo IFN-gamma production by CD8 T cells during infection. J. Immunol..

[74] McSorley HJ, Blair NF, Smith KA, McKenzie AN, Maizels RM (2014). Blockade of IL-33 release and suppression of type 2 innate lymphoid cell responses by helminth secreted products in airway allergy. Mucosal Immunol..

[75] Kwock JT (2020). IL-27 signaling activates skin cells to induce innate antiviral proteins and protects against Zika virus infection. Sci. Adv..

[76] Hisada M (2004). Potent antitumor activity of interleukin-27. Cancer Res..

[77] Salcedo R (2004). IL-27 mediates complete regression of orthotopic primary and metastatic murine neuroblastoma tumors: role for CD8+ T cells. J. Immunol..

[78] Matsui M (2009). Interleukin-27 activates natural killer cells and suppresses NK-resistant head and neck squamous cell carcinoma through inducing antibody-dependent cellular cytotoxicity. Cancer Res..

[79] Chiba Y (2018). Interleukin-27 exerts its antitumor effects by promoting differentiation of hematopoietic stem cells to M1 macrophages. Cancer Res..

[80] Yoshimoto T (2008). Antiproliferative activity of IL-27 on melanoma. J. Immunol..

[81] Zorzoli A (2012). Interleukin-27 inhibits the growth of pediatric acute myeloid leukemia in NOD/SCID/Il2rg-/- mice. Clin. Cancer Res..

[82] Di Carlo E (2014). The antitumor potential of interleukin-27 in prostate cancer. Oncotarget.

[83] Cocco C (2010). Interleukin-27 acts as multifunctional antitumor agent in multiple myeloma. Clin. Cancer Res..

[84] Airoldi I (2015). Interleukin-27 re-educates intratumoral myeloid cells and down-regulates stemness genes in non-small cell lung cancer. Oncotarget.

[85] Chiba Y (2013). IL-27 enhances the expression of TRAIL and TLR3 in human melanomas and inhibits their tumor growth in cooperation with a TLR3 agonist poly(I:C) partly in a TRAIL-dependent manner. PLoS ONE.

[86] Oft M (2014). IL-10: master switch from tumor-promoting inflammation to antitumor immunity. Cancer Immunol. Res..

[87] Karakhanova S, Bedke T, Enk AH, Mahnke K (2011). IL-27 renders DC immunosuppressive by induction of B7-H1. J. Leukoc. Biol..

[88] Carbotti G (2013). The IL-18 antagonist IL-18-binding protein is produced in the human ovarian cancer microenvironment. Clin. Cancer Res..

[89] Zhai L (2020). Immunosuppressive IDO in cancer: mechanisms of action, animal models, and targeting strategies. Front. Immunol..

[90] Carbotti G (2015). IL-27 induces the expression of IDO and PD-L1 in human cancer cells. Oncotarget.

[91] Jia H, Dilger P, Bird C, Wadhwa M (2016). IL-27 promotes proliferation of human leukemic cell lines through the MAPK/ERK signaling pathway and suppresses sensitivity to chemotherapeutic drugs. J. Interferon Cytokine Res..

[92] Di Meo S (2014). Interleukin-30 expression in prostate cancer and its draining lymph nodes correlates with advanced grade and stage. Clin. Cancer Res..

[93] Sorrentino C (2018). Interleukin-30/IL27p28 shapes prostate cancer stem-like cell behavior and is critical for tumor onset and metastasization. Cancer Res..

[94] Airoldi I (2016). Interleukin-30 promotes breast cancer growth and progression. Cancer Res..

[95] Pflanz S (2002). IL-27, a heterodimeric cytokine composed of EBI3 and p28 protein, induces proliferation of naive CD4(+) T cells. Immunity.

[96] Muller SI, Aschenbrenner I, Zacharias M, Feige MJ (2019). An interspecies analysis reveals molecular construction principles of interleukin 27. J. Mol. Biol..

[97] Rousseau F (2010). IL-27 structural analysis demonstrates similarities with ciliary neurotrophic factor (CNTF) and leads to the identification of antagonistic variants. Proc. Natl Acad. Sci. USA.

[98] Feige, M. J. *Oxidative Folding of Proteins: Basic Principles, Cellular Regulation and Engineering*. (RSC Publishing, 2018).

[99] Yoon C (2000). Charged residues dominate a unique interlocking topography in the heterodimeric cytokine interleukin-12. EMBO J..

[100] Posadas-Sanchez R (2017). Interleukin-27 polymorphisms are associated with premature coronary artery disease and metabolic parameters in the Mexican population: the genetics of atherosclerotic disease (GEA) Mexican study. Oncotarget.

[101] Zhou B (2015). Polymorphisms and plasma levels of IL-27: impact on genetic susceptibility and clinical outcome of bladder cancer. BMC Cancer.

[102] Tang YJ (2014). Associations of IL-27 polymorphisms and serum IL-27p28 levels with osteosarcoma risk. Medicines.

[103] Chae SC (2007). Identification of polymorphisms in human interleukin-27 and their association with asthma in a Korean population. J. Hum. Genet..

[104] He W (2019). Association of single-nucleotide polymorphisms in the IL27 gene with autoimmune thyroid diseases. Endocr. Connect.

[105] Li CS (2009). Interleukin-27 polymorphisms are associated with inflammatory bowel diseases in a Korean population. J. Gastroenterol. Hepatol..

